# Carbonyl-protein content increases in brain and blood of female rats after chronic oxycodone treatment

**DOI:** 10.1186/s12868-020-0552-2

**Published:** 2020-01-22

**Authors:** Ruping Fan, Lisa M. Schrott, Stephen Snelling, John Felty, Derrel Graham, Patrick L. McGauly, Thomas Arnold, Nadejda L. Korneeva

**Affiliations:** 1 Department of Emergency Medicine, Louisiana State University Health Sciences Center, 1501 Kings Highway, Shreveport, USA; 2 Department of Pharmacology, Toxicology, and Neuroscience, Louisiana State University Health Sciences Center, 1501 Kings Highway, Shreveport, USA

**Keywords:** Oxycodone, Opioid, Cortex, Oxidative stress, Integrated stress response, Carbonyl-protein, Carbonylation, Protein aggregates

## Abstract

**Background:**

Opioids are the most effective drugs commonly prescribed to treat pain. Due to their addictive nature, opioid pain relievers are now second to marijuana, ahead of cocaine with respect to dependence. Ours and other studies suggest potential toxic effects of chronic opioid administration leading to neuronal degeneration. It has been suggested that protein carbonylation may represent a sensitive biomarker of cellular degeneration. To evaluate whether prolonged oxycodone administration is associated with accumulation of protein aggregates that may contribute to neuronal degeneration we measured protein carbonylation levels in brain and also in blood plasma of rats after 30-days of 15 mg/kg daily oxycodone administration.

**Results:**

We observed a significant increase in the level of carbonylated proteins in rat brain cortex after 30-days of oxycodone treatment compare to that in water treated animals. Also, oxycodone treated rats demonstrated accumulation of insoluble carbonyl-protein aggregates in blood plasma.

**Conclusions:**

Our data suggests that tests detecting insoluble carbonyl-protein aggregates in blood may serve as an inexpensive and minimally invasive method to monitor neuronal degeneration in patients with a history of chronic opioid use. Such methods could be used to detect toxic side effects of other medications and monitor progression of aging and neurodegenerative diseases.

## Background

Opioids are a type of drugs that commonly prescribed to treat pain. Due to their addictive nature, opioid pain relievers are now second to marijuana, ahead of cocaine with respect to dependence. According to a recent study, more than 10 percent of cancer patients continue to take opioids for several months after surgery, far longer than it is clinically recommended [1]. In 2010, a study investigating the effect of prescription opioids on brain structure of opioid dependent patients demonstrated the correlation between duration of drug use and alternations in brain functional connectivity, especially in regions responsible for impulse control, reward and motivation [2]. These changes included leukoencephalopathy, axon demyelination, and white matter lesions. Thus, it is important to monitor effect of opioid therapy on brain health status. The main methods to monitor neuronal degeneration in the brains of living people are the magnetic resonance imaging (MRI) and Positron Emission Tomography (PET) scans. In addition, measuring toxic protein aggregates in cerebrospinal fluid from a lumbar puncture of Alzheimer’s disease patient is another method to confirm development of neuronal degeneration. However, MRI and PET scan are expensive procedures that are typically not covered by insurance. Spinal taps are invasive and may lead to health complications. Thus, development of an inexpensive and minimally invasive method is important to monitor neuronal degeneration in patients with a history of chronic opioid use. Such method could also be used to detect toxic side effects of other medication and monitor progression of aging and neurodegenerative diseases.

In general, the rate of degeneration of an individual organism depends on the cells ability to balance production of newly synthesized molecules and disposal of toxic waste. The events leading to cellular waste accumulation, in the form of protein aggregation, include oxidative stress, decreased proteasomal activity and reduced autophagy. Protein aggregation eventually manifests in cell functional decline, degeneration and apoptosis. Accumulation of protein aggregates is a common biological phenomenon, observed in different physiological and pathological conditions. Protein modification, such as carbonylation, causes major changes in protein structure and function leading to formation of protease resistant protein aggregates, which result in the loss of cell viability (reviewed in [3]). Several studies have shown also that *protein carbonylation* increases proportionately with increasing age of cells, organelles, and tissues in diverse species [3, 4]. Recently, an increase in protein carbonyl content was associated with development of neurodegenerative diseases such as Alzheimer’s and Parkinson’s diseases, and also with cancer, cataractogenesis, atherosclerosis, diabetes, sepsis and aging (reviewed in [4]).

Ours and other studies have shown that chronic opioid administration is associated with activation of the pro-apoptotic signaling and neuronal degeneration in animal models [5–9]. In our current study, we analyzed carbonyl content in brain and blood/plasma samples from the same animals that have been used to evaluate oxidative and neurodegenerative effect of oxycodone reported in [5, 10]. We demonstrated increased levels of protein carbonylation in rat cortex and also accumulation of Triton™ X-100 insoluble carbonyl-protein aggregates in blood plasma of animals treated with oxycodone, indicating a systemic degenerative process. Moreover, we developed a method to detect insoluble carbonylated protein aggregates in rat plasma that, we suggest, may be applied as a detection method of neuronal degeneration.

## Methods

### Animal model and tissue preparation

In this study, we have used tissue samples from female 60 day-old Sprague–Dawley rats that have been reported in our previous studies [5, 10]. Briefly, randomly assigned animals were gavaged with vehicle water or with 15 mg/kg oxycodone (Mallinckrodt Inc., St. Louis MO) in a volume of 1.0 ml/kg every 24 h for 30 days. Lack of toxicity and efficient anti-nociceptive effect of this oxycodone scheme treatment were assessed by daily weight measurement and by the hot plate tests, respectively, as it is described in [5, 10]. We investigated tissues from twelve water and twenty oxycodone treated rats using from four sets of littermates. Experiments # 1, 2, and 3 contained nine littermates each: three rats were gavaged with water and six animals gavaged with oxycodone. Experiment # 4 contained five littermates: three rats treated with water and two rats treated with oxycodone. In the experiments # 1, 2, and 3, brain tissues containing specific areas were pooled together from three rats in the same treatment group yielding one water brain lysate (W) and two oxycodone brain lysates (O1 and O2) for each experiment. In the experiment #4, we analyzed brain lysate from individual animals (W1, W2, W3, O1 and O2). Brain lysate preparation is described in our earlier study [5]. Similarly, blood and plasma samples were prepared from pooled three corresponding rat samples in the same treatment group in the experiments # 1, 2, and 3, and from individual samples in the experiment # 4.

### Immunofluorescent staining of carbonylated proteins in rat brain

Brain tissue preparation and immunofluorescent staining procedure were described elsewhere [5]. Briefly, in each experiment, water and oxycodone treated rats have been sacrificed on the same day by injection of 65 mg/kg i.p. of sodium pentobarbital and perfusion with ice-cold saline followed by 4% paraformaldehyde in 0.1 M sodium phosphate buffer, pH 7.4. The whole brains were stored in 70% ethanol at 4 °C until further processing. Slides containing 10 µm thick paraffin slices of cortex sections (plates 12–30, Rat Brain Atlas, Paxinos and Watson) were prepared as it is described in [5]. After deparaffinization, slides were incubated with 2,4-dinitrophenylhydrazine (DNPH) solution for 15 min at RT, blocked with horse serum for 1 h and then incubated with anti-DNP antibodies (dilution 1:2000) in humidified chamber overnight at 4 °C. Next morning, after wash with TBS-T (20 mM Tris–HCl, 150 mM NaCl, and 0.1% Tween® 20, pH 7.5), slides were incubated with biotin-conjugated anti-goat IgG (dilution 1:200, Santa Cruz, cat # sc-2042) for 2 h at RT and then with streptavidin conjugated-AlexaFlour®-594 (dilution 1:200, Life Technologies, cat # S11227) for one hour at RT. Each brain slice was covered by VECTASHIELD HardSet Antifade Mounting Medium with DAPI (Vector Laboratories Inc., H-1500) and a cover slip. Images were visualized using AxioObserver with ApoTome microscope (Zeiss) as it is described in [5].

### BioVision assay

Protein carbonyl groups in rat brain lysates were measured by the Protein Carbonyl Content Assay Kit (BioVision, Inc.) according to the manufacture’s protocol. Briefly, one hundred microliters of cortex lysates with concentration 10 mg/ml were combined with 100 µl DNPH, vortexed and incubated for 10 min at room temperature (RT). Proteins were precipitated with 30 µl trichloroacetic acid by centrifugation the samples at 20,000×*g* at 4 °C for 2 min. Pellets were washed with cold acetone (− 20 °C), resuspended in 200 µl guanidine solution, briefly sonicated and then incubated at 60 °C for 30 min. One hundred microliters aliquots were transferred to 96-well plate. Absorbance was measured at 375 nm in a microplate reader. Other five microliters aliquots were used to measure protein concentration in each sample by Micro BCA™ Protein Assay Kit (Thermo Scientific, Pierce) using BSA as a standard.

### Blood collection and plasma preparation

Blood samples were collected from the same animals used for the brain lysate preparation described above. In the experiments #1, 2, and 3, each blood collection tube contained blood from three animals. For example, in the experiment #1, the tube labeled “W” contained blood from three rats treated with water, “O1”—blood from three rats treated with oxycodone; and “O2”—another three rates treated with oxycodone. Total, the blood from 9 animals was used in the experiment #1 (3 water and 6 oxycodone samples). Similarly, in the experiments #2 and 3, the blood from 9 animals was used in each experiment. In the experiment #4, each blood collecting tube contained blood from one animal. Thus, each tube labeled W1, W2, and W3 contained blood from one animal treated with water. Each tube labeled O1 and O2 contained blood from one animal treated with oxycodone. Blood samples were collected in conical tubes containing 2 ml buffer A per rat, supplemented with 0.1 mg/ml cycloheximide, 10 mg/ml heparin, Complete™ EDTA-free protease inhibitor cocktail (Roche) and phosphatase inhibitor cocktails (Pierce Biotechnology), on ice. Cells and plasma were separated by centrifugation at 1000 ×*rpm* at 4 °C for 5 min. The supernatants (plasma) were collected and then protein concentration was determined using Micro BCA™ Protein Assay Kit.

### Dot-blot assay

For dot blot analysis of protein carbonylation in rat blood plasma, samples containing twenty five to thirty micrograms of protein were incubated with equal volume of DNPH solution for 15 min at RT. After neutralization with Tris-Glycerol solution (2 M Tris and 30% Glycerol), two microliters aliquots were spotted on a Nitrocellulose membrane (Bio-Rad) and then analyzed by incubation with anti-DNP antibodies (dilution 1:1000; Fitzgerald, 20R-DG001) overnight at 4 °C and then with anti-goat secondary antibodies (dilution 1:5,000) conjugated with horse-peroxidase (Vector Laboratories, Inc.) in 5% non-fat dry milk in TBS-T. Blots were developed as it is described in [5] using the Western Lightning ECL Pro development kit (PerkinElmer) and were exposed to HyBlot CL autoradiography film (Denville Scientific). The ImageQuant TL software (GE Healthcare Life Science) was used for quantitative analysis of dot blot images.

### Protein aggregation assay

Aliquots containing eight hundred micrograms of total plasma protein were centrifuged at 20,000×*g* at 4 °C for 30 min. Pellets were resuspended in 50 µl buffer C (50 mM Tris–HCl, pH 8.0, 100 mM KCl) supplemented with 1% Triton™ X-100, incubated on ice for 15 min and centrifuged again at 20,000×*g* at 4 °C for 30 min. Resulting pellets were resuspended in 10 µl buffer C supplemented with SDS. Five microliters aliquots of resuspended pellets were incubated with 5 µl DNPH reagent for 15 min at RT. Reactions were neutralized by adding 3 µl Tris-Glycerol solution. Two microliters aliquots were then spotted on a Nitrocellulose membrane and then analyzed by anti-DNP antibodies as it is described in the Dot-blot assay section.

### Western blot analysis

The western blot analysis was performed as it is described in [5, 10]. Briefly, to analyse the expression of proteins in blood, equal amounts of total protein were loaded on a 12% or 4–12% NuPAGE® Novex® Bis–Tris Gel (Invitrogen). To identify the position of specific proteins the Full-Range Rainbow protein molecular weight marker (GE Healthcare Life Science) was loaded on the same gel. After separation by the SDS-PAGE gel proteins were transferred to a Nitrocellulose membrane (Bio-Rad) using a Mini Trans-Blot cell (Bio-Rad). Membranes were incubated with primary antibodies: anti-P-eIF2α (Ser51) (dilution 1:2000) and anti-eIF2α (D7D3) XP (dilution 1:4000), all from Cell Signalling, at 4 °C for overnight. Anti-rabbit secondary antibodies conjugated with horse-peroxidase (Vector Laboratories, Inc.) were diluted to 1:20,000. Signal detection and analysis were performed as it is described in [5, 10] using Western Lightning ECL Pro development kit (PerkinElmer), HyBlot CL autoradiography film (Denville Scientific) and the ImageQuant TL software (GE Healthcare Life Science). Results are presented as the mean of at least three independent treatment (drug administration) experiments ± SEM.

### Statistical analyses

The experiments were analysed blindly. All data and are presented as treatment means ± SEMs (n ≥ 3). Statistical analysis was performed using a Student’s t-test and one-way analysis of variance test ANOVA following Tukey’s ranking tests. Data with p value lower than 0.05 was considered to be statistically different.

## Results

### Oxycodone increases protein carbonyl content in rat cortex

**Fig. 1:**
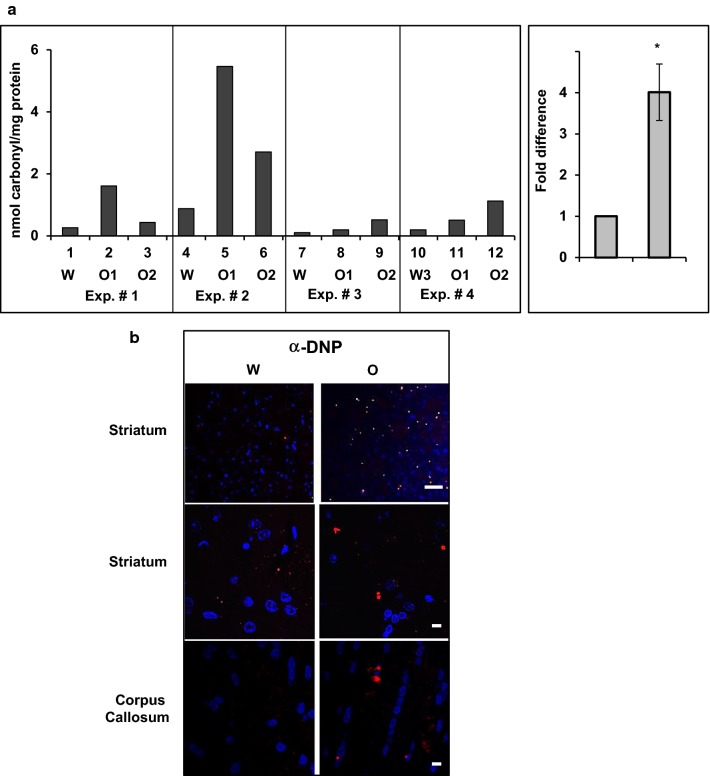
Chronic oxycodone treatment increases protein carbonyl content in rat cortex. **a** BioVision carbonyl content assay of proteins from cortex lysates of rats treated with water (W) or oxycodone (O1, O2). Left panel, assay was performed according to a manufacture protocol using four sets of animal groups that represented four different litters, labeled as Exp. #. Data expressed as nanomol of carbonyl groups per milligram of protein using BSA as standard. Right panel, graph representing mean value of carbonyl content in cortex lysates normalized to the corresponding water samples set as one for each experiment (± SEM; n = 4 (four BioVision experiments that analyzed samples from 10 water and 20 oxycodone treated rats; p = 0.012). Statistical analysis of carbonyl content in oxycodone relative to water exposed cortex lysates was performed using Student’s t-test. **b** Immunofluorescent analysis of carbonylated proteins in cortex of rats treated with water (W) or oxycodone (O). Scale bar denotes 50 µm for the top striatum images and 5 µm for the middle striatum and lower corpus callosum images

To investigate whether chronic opioid administration increases the level of carbonyl-protein content in rat brain, we analyzed brain lysates of rats orally gavaged with 15 mg/kg of oxycodone every 24 h for 30 days. This study involves the same set of animals reported earlier [5, 10]. The BioVision assay of brain lysates from nucleus accumbens, cortex and cerebellum revealed a significant increase in carbonyl content only in cortex lysates of oxycodone treated animals. Analysis of cortex lysates of rats from four different litters representing four different experiments demonstrated that, although the base level of protein carbonylation (water-treated animals) varied significantly between different rat litters, the relative increase in carbonyl signal in oxycodone-exposed tissues compared to that in water-treated rats in the same litter was reproducible in all four different experiments (Fig. 1a). On average, chronic oxycodone treatment increased the carbonyl-protein levels in rat brain tissues by 4.01 ± 0.69 fold (p = 0.012) compare to that in water treated animals (Fig. 1a, right graph). We then investigated localization of carbonylated proteins in cortex areas of rats treated with water or oxycodone by immunofluorescent staining of brain slices derivatized with 2,4-dinitrophenylhydrazine (DNPH) (Fig. 1b). We observed increased numbers of DNP-labelled specks throughout of the cortex of oxycodone-exposed animals (14 to more than 50 specks per field) compared to that in water-treated cortex samples (0 to 5 specks per field) (Fig. 1b, upper images). Moreover, the size of such DNP-labelled specks also increased in oxycodone brain tissues (Fig. 1b, middle and lower images). Altogether, these data suggest that prolonged oxycodone administration is associated with accumulation of carbonyl-protein content in rat brain.

### Oxycodone induces insoluble protein aggregates in rat blood plasma

**Fig. 2:**
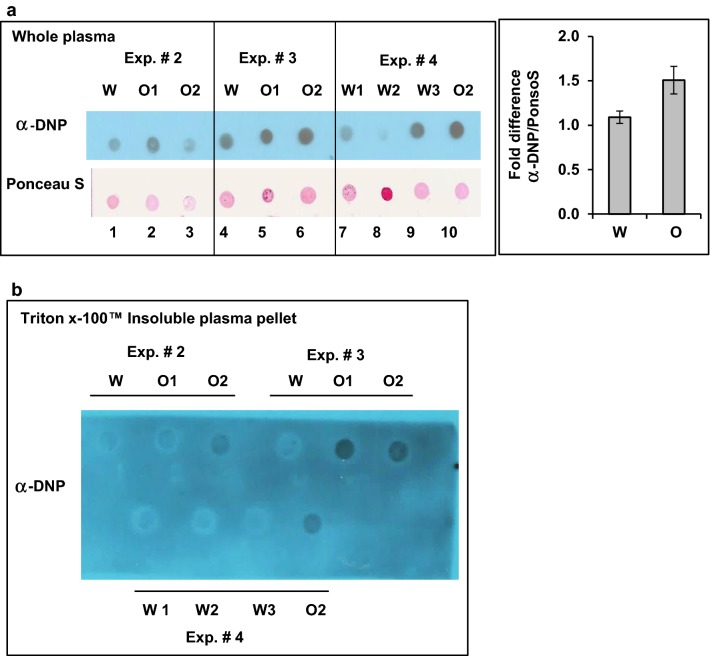
Chronic oxycodone treatment increases protein carbonyl content in rat plasma. **a** Dot-blot analysis of carbonylated proteins in rat plasma. Left panel, equal amount of total protein from plasma of rats treated with oxycodone (O1, O2) or water (W) were derivatized with DNPH, spotted on NC membrane and probed with anti-DNP antibodies. The same membrane was later stained with Ponceau S to detect total protein in each spot. Each plasma sample corresponds to the same animal from which cortex samples was obtained (Fig. 1a, Exp. #). The corresponding experiment is indicated above each set of samples. Right graph, quantitative analysis of dot-blot images shown on left. DNP signal was normalized to Ponceau S signal in corresponding sample and then oxycodone value was normalized to water value in the same experiment. Graph represents mean value of DNP to Ponceau S ratio normalized to water samples set as one (± SEM; n = 3 (three sets of experiments that analyzed samples from 9 water and 13 oxycodone treated rats; p = 0.1). **b** Dot-blot analysis of Triton™ X-100 insoluble carbonylated proteins in rat plasma. Equal volume of plasma samples from rats treated with water (W) or oxycodone (O) corresponding to samples presented in **a** was centrifuged at 20,000×*g* for 30 min. The pellets were resuspended in buffer containing 1% Triton™ X-100 and centrifuged again. Resulting pellets were dissolved in buffer, derivatized with DNPH, spotted on NC membrane and probed with anti-DNP antibodies

Next, we investigated whether the changes in carbonyl-protein content in the brain would correlate with the changes in blood plasma obtained from the same animals. Equal aliquots of plasma samples from the same animals that were tested for carbonyl content in the cortex (Fig. 1a) were derivatized, spotted on the nitrocellulose (NC) membrane and probed with antibodies against DNP (Fig. 2a, upper image) or stained with Ponceau S to detect the level of total protein in each spot (lower image). Within each experiment (representing the same litter), oxycodone-exposed plasma samples had higher carbonyl-protein content. However, statistical analysis of all three experiments showing about 1.51 ± 0.16 fold (p = 0.1) increase in carbonyl content in oxycodone-exposed plasma compared to that in water-treated animals did not reach statistical significance (Fig. 2a, right graph).

In general, an increase in protein carbonyl content does not necessarily lead to cell death and degeneration. Carbonylation may act as a signal for protein degradation since carbonylated proteins are more susceptible to proteolytic degradation. It is only when heavily carbonylated proteins start to form aggregates that are resistant to degradation that they become toxic and can lead to cell death. Thus, we investigated whether chronic oxycodone administration is associated with accumulation of Triton™ X-100 insoluble protein aggregates in plasma. Equal aliquots of plasma samples from the same animals as shown on Fig. 2a were centrifuged at 20,000×*g* for 30 min. The pellets were resuspended in a buffer containing 1% Triton™ X-100 and centrifuged again. Resulting pellets were dissolved in a buffer containing SDS, derivatized with DNPH and analysed by dot-blot (Fig. 2b). Remarkably, in all three experiments, dot-blot analysis showed a more than 2.8-fold statistically significant (p = 0.017) increase in carbonyl-protein content in the Triton™ X-100 insoluble pellets from the oxycodone-exposed samples compared to that from the water-treated animals (Fig. 2b). These data suggest that chronic oxycodone exposure is associated with accumulation of Triton™ X-100 insoluble carbonylated proteins in blood plasma.

### Oxycodone induces the integrated stress response in rat blood

**Fig. 3:**
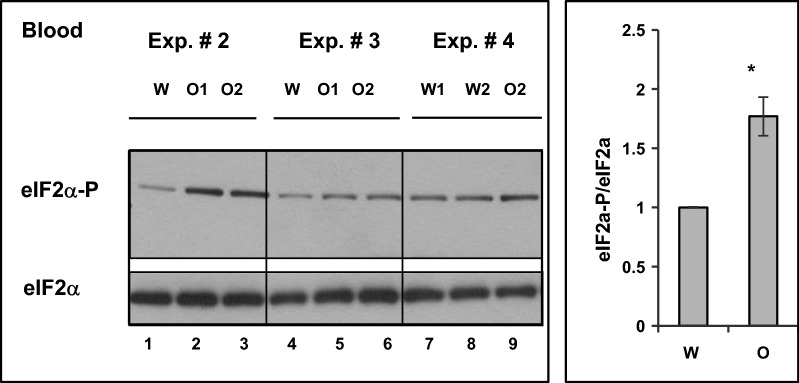
Chronic oxycodone treatment activates the Integrated Stress Response in rat blood. Western blot analysis of phosphorylated eIF2α in rat blood. Left panel, equal amount of total protein from blood of rats treated with oxycodone (O1, O2) or water (W) corresponding to samples presented in Fig. 2a. was analysed by western blot probed with anti-phospho eIF2α and total eIF2α. Right panel, graph representing mean value of phospho-eIF2α to total eIF2α ratio normalized to that in water samples set as one (± SEM; n = 4 water and n = 5 oxycodone (three sets of experiments that analyzed samples from 8 water and 13 oxycodone treated rats; p = 0.048). Statistical analysis of carbonyl content in oxycodone relative to water exposed cortex lysates was performed using Student’s t-test

In our previous study we demonstrated that chronic oxycodone administration is associated with activation of pro-apoptotic signaling, loss of white matter, reactivation of astrocytes [5] and induction of the integrated stress response (ISR) in rat brains [10]. In our current study of the tissues samples from the same animals, we observed an increase in the level of carbonyl-protein content in brain and blood plasma of oxycodone treated rats. What could trigger the neurotoxic signaling during prolonged oxycodone exposure? The ISR *triggers* a general adaption mechanism that provides necessary support for cells to survive during stress and also promotes recovery. However, under prolong stress conditions, high expression of the ISR transcription factors ATF4 (cAMP element binding transcription factor 4 or activating transcription factor 4) and CHOP (DNA damage/C/EBP homology protein) may lead to increase in protein synthesis and induce an accumulation of large molecular protein complexes, ATP depletion, oxidative stress and cell death [11–13]. To evaluate whether accumulation of carbonyl-protein aggregates correlates with induction of the ISR in blood we monitored the level of phosphorylated translation initiation factor 2 alpha (eIF2α) in blood samples from the same animals (Fig. 3). Western-blot analysis showed almost 1.77 ± 0.16 fold statistically significant (p = 0.048) increase in phospho-eIF2α in oxycodone blood samples compared to that in water sample. These data suggest that prolonged activation of the ISR during opioid exposure may contribute to accumulation of carbonylated protein aggregates that eventually may lead to cellular degeneration.

## Discussion

While the identification of biomarkers of neurodegeneration is critical to the early detection and treatment of adverse drug effects, currently there is no specific blood test to monitor the rate of degenerative disease progression at the level of the individual organism. The blood protein carbonylation assay may serve this purpose. It has been suggested that protein carbonylation may represent a sensitive biomarker of cellular degeneration due to excessive stress, aging, or certain pathologies [4] including Alzheimer’s disease [14]. However, studies monitoring carbonyl protein content in clinical settings produced contradictory results. The reason for lack of “the protein carbonylation as a biomarker of degeneration” test on a market is that most of the studies have been measuring total (soluble and insoluble) carbonyl content in tissues or body fluids that may produce a false positive result and not always correlate with the degree of degeneration. In general, increase in protein carbonyl content does not necessarily lead to cell death and degeneration. It is only when excessive carbonyl content induces significant changes in protein structure leading to formation of protease resistant protein aggregates that become toxic and may lead to cell death (reviewed in [3]). Our data suggest that carbonyl protein aggregates may serve as a reliable biomarker of degeneration.

Previously we documented that chronic oxycodone administration associated with axonal degeneration and induction of oxidative and nitrosidative stresses and activation of the integrated stress response (ISR) in rat brain [5, 10]. Since opioid abuse is associated with hypoxia, endoplasmic reticulum stress and the induction of excitotoxic stress, we have hypothesised that opioids cause systemic stress on the whole organism. Indeed, in current study we observed activation of the ISR in blood of animals chronically treated with oxycodone. The integrated stress response is a general mechanism that allows cells to adapt to various types of stresses [15]. The key event in the ISR, regardless of the trigger, is phosphorylation of eIF2α that modulates expression and translational activation of specific mRNAs, such as ATF4 and CHOP, which determine whether the cell will adapt to the stress condition or undergo to apoptosis. However, under prolong stress conditions, elevated ATF4 and CHOP expression increases protein synthesis and thus causes accumulation of high molecular protein complexes, ATP depletion, oxidative stress and cell death [11–13]. Elevated eIF2α phosphorylation was correlated with neuronal degeneration and was observed in the brain samples of Alzheimer’s disease (AD) patients [16–19]. Thus, it is possible that prolonged activation of the ISR during opioid exposure results in accumulation of carbonylated protein aggregates that eventually lead to neuronal degeneration.

In our current study, we observed increase in carbonylated proteins in cortex areas and also accumulation of Triton X-100-insoluble carbonylated protein aggregates in rat blood plasma after prolonged oxycodone treatment. Recently, protein carbonylation and aggregation was linked to neuronal cell death in an animal model of experimental autoimmune encephalomyelitis characterized by central nervous system inflammation, demyelination and axonal degeneration [20].

## Conclusions

Our data suggest that measuring amount of carbonyl-protein content in insoluble fraction of blood may serve as a tool to monitor the rate of neuronal degeneration and degeneration in general in individual patient. Moreover, identification of unique proteins in these protein insoluble aggregates may facilitate development of a new diagnostic tool. This approach will help physicians and patients to evaluate current progression of the disease and efficiency of the therapy.

## Data Availability

The datasets used and/or analyzed during the current study available from the corresponding author on reasonable request.

## Abbreviations

ATF4: cAMP element binding transcription factor 4 or activating transcription factor 4
BSA: bovine serum albumin
CHOP: DNA damage/C/EBP homology protein, GADD153
CNS: central nervous system
DNPH: 2,4-dinitrophenylhydrazine
ISR: integrated stress response
NC: nitrocellulose
Oxy: oxycodone
RT: room temperature
W: water
